# Time trend of neurocysticercosis in children with seizures in a tertiary hospital of western Nepal

**DOI:** 10.1371/journal.pntd.0005605

**Published:** 2017-05-10

**Authors:** Kalipatnam Seshagiri Rao, Sudhir Adhikari, Eva Gauchan, Brijesh Sathian, Ganesh B. K., Sahisnuta Basnet, Prabhat Kumar Tiwari, Namraj Bahadur, Rajnish Mishra

**Affiliations:** 1 Dept of Pediatrics, Manipal Teaching Hospital, Pokhara, Nepal; 2 Dept of Community Medicine, Manipal Teaching Hospital, Pokhara, Nepal; 3 Dept of Radiodiagnosis, Manipal Teaching Hospital, Pokhara, Nepal; Universidad Nacional Autónoma de México, MEXICO

## Abstract

**Introduction:**

Neurocysticercosis is a common cause of seizure disorders in children of Western Nepal. The clinical presentation is variable. The incidence varies depending on the food habits and ethnicity of the population. The present study was undertaken with the objective of studying the mode of presentation, radiological findings and to determine the recent trend of the disease in children of Western Nepal.

**Methods:**

Records from the Department of Pediatrics, Manipal Teaching Hospital, Pokhara, Nepal of children aged 0–17 years admitted from 2003 to 2015 and with the discharge diagnosis of seizure and neurocysticercosis (NCC) were reviewed. The diagnosis was primarily based on clinical features, neurological involvement and CT and MRI studies. Seizures due to other CNS pathologies were excluded. Patients with NCC were treated with Albendazole15mg/kg/day for 28 days with supportive treatments for seizures and raised intracranial pressure. Patients were followed up for one year after the completion of the treatment.

**Results:**

There were 1355 cases of seizure disorders, out of which 229 (16.90%) were NCC. There were 99 (43.23%) in the age group 6–10 years followed by 91 (41.09%) in the age group of 11–15 years. Seizures were the most common presenting symptom in 88.65%, followed by raised ICP in 9.61%. Neuropsychiatric changes were noted in 38 cases (16.59%). CT scan findings revealed single lesion in 78.16% and multiple lesions in 21.83%. Poisson regression analysis showed statistically significant decline of year-wise incidence of NCC cases (p<0.05) from 2003 to 2015.

**Conclusion:**

The decline in the incidence of NCC in recent years is most probably attributed to improved hygiene with the construction of household toilets to avoid open defecation and biannual deworming with Albendazole as a part of School Health and Nutrition Project.

## Author summary

Neurocysticercosis is a common parasitic infection of the central nervous system. It is caused by larval form of *Taenia solium* and it has been identified as a “Neglected Tropical Disease” endemic in south East Asia, including Nepal, by WHO. The clinical features in children are pleomorphic depending on the number, location and size of cysticerci. The parenchymatous lesions are presented as neurological symptoms like sudden onset seizures, encephalitis, raised intracranial pressure and neuropsychiatric symptoms. CT and MRI are the main diagnostic tools. Maximum incidence was found in school age children (6–15 years). Year wise review of percentage of NCC, revealed a declining trend from 20.83% in 2009 to 5.37% in 2014. The decline in the incidence of NCC noted in our study might be due to biannual deworming using Albendazole as a part of School Health and Nutrition Programme started by Ministry of Health Services, Nepal in 2008 and extended to all the districts of the country. In addition construction of household and school toilets to avoid open defecation, health education and hand washing facilities might have played a role. Large multicentre trials to evaluate role of biannual Albendazole to prevent cysticercosis is recommended.

## Introduction

Neurocysticercosis (NCC) or intracerebral cysticercosis is a common parasitic (zoonotic) infection involving the nervous system of human beings resulting in seizure disorder worldwide [1,2]. NCC is endemic in parts of South America, Eastern Europe, the Indian subcontinent, South East Asia and Sub-Saharan Africa [2]. NCC is an important cause of focal seizures in north India [3]. Human and porcine taeniasis/NCC is a major zoonotic disorder in Nepal [4–6]. Prevalence rates of NCC in children of Nepal is not known, although in certain ethnic groups adult taeniasis was reported to be 10–50% and porcine cysticercosis 14 to 32% [4–6].

The cyst has four stages: *Vesicular stage*—The scolex is on one side of cyst and appears as an opaque 4 to 5 mm nodule. Once the cyst degenerates an inflammatory response is elicited and goes through a colloidal stage where larva undergoe hyaline degeneration and gelatinous material appears in the cyst. *Granular nodular stage-* the cyst contracts and is replaced by local lymphoid nodules and necrosis. *Nodular calcified stage*- the granular tissue is replaced by collagenous structure and calcification. NCC involves the brain parenchyma, meninges, spinal cord and eyes. Cysticerci also develop in muscles. Clinical manifestations mainly depend on the location, numbers and variability of the cysts and on the host immune response [7].

The most common CT finding in children presenting with seizures is a single small (<20mm), low density lesion with ring enhancement named Single Small Enhancing Computer Tomographic Lesion. It represents a cystic lesion associated with mild to moderate perilesional edema and hyperdense eccentric scolex which is pathognomonic of cysticercosis. Numerous cysts of varying stages give the typical ‘Starry Sky” appearance [8].

NCC is one of the main causes of epileptic seizure and accounts for 50% of the patient with partial seizures [9,10]. In an earlier study conducted at the Manipal Teaching Hospital, Pokhara, a tertiary care center of western Nepal, 109 patients (16.07%) were diagnosed as cases of NCC amongst 678 cases admitted for partial seizures in a period extending from 2004 to 2009 [11]. The incidence of NCC among people admitted for epilepsy in Latin America, sub Saharan Africa and south East Asia is 29% [12]. The larvae develop in brain parenchyma, meninges, spinal cord, eyes and muscles, and will result in symptoms. Parenchymatous lesions usually manifest as seizure disorder or epilepsy, being simple partial seizures, complex partial seizures, generalized seizure, encephalitis and pseudotumor cerebri. Extra parenchymal NCC is less common in children compared to adults and may appear as multilobed CSF isointense lesions that occupy the cisterns, Sylvian fissure or cerebellopontine angle. Arachnoiditis and chronic meningitis, such as enhancement of tentorium and basal cisterns, hydrocephalous and occasionally infarcts may be seen [13]. With such variability in incidence and clinical presentation of NCC all over the world, we attempted to review the documents of all seizure disorders admitted and discharged of the Manipal Teaching Hospital with diagnosis of NCC from January1^st^ 2003 to December 31^st^ 2015.

## Materials and methods

This study was conducted in the department of Pediatrics, Manipal Teaching Hospital, Pokhara, Nepal located at the western region of Nepal from 2003 to 2015 a period of 13 years. Hospital records of pediatric patients between 0 to 17 years admitted with seizure disorder were reviewed for diagnosis of NCC. The diagnosis was primarily based on clinical features and CT. MRI was available from January 2009 for the confirmation of doubtful findings of NCC by CT. The following features are considered NCC: vesicular cysts generally with hypodense fluid; non-enhancing or mildly enhancing lesions not surrounded by edema; degenerating cysts appear as small isodense lesions with ring enhancement after contrast medium, the scolex appears as a bright high density eccentric nodule that is pathognomonic of NCC; calcified cysts are small, single or multiple and generally without edema. Children with active seizures may show edema around calcified lesions. MRI was considered in extra parenchymal lesions. Scolices and living cysticerci are round, either isometric or slightly hyper-intense to the cerebrospinal fluid (CSF).

Electroencephalography (EEG) for seizures, fundus examination for ophthalmological involvement and stool examination for tapeworm eggs or proglottids were carried out when necessary. Patients with seizures not showing evidences of NCC with history of asphyxia neonatorum, neonatal hypoglycemia, hyper-bilirubinemia, birth trauma, congenital abnormalities with neurodevelopmental delay and cerebral palsy were not included in the study.

Patients were treated following defined protocols; Albendazole at 15mg/kg/day in divided dosage for 28 days and antiepileptic medication for 2 years after seizure free. Also, intravenous dexamethasone was given at 0.5mg/kg/day in 3 divided dosages for 3 days before starting treatment with Albendazole. When marked evidence of raised intracranial pressure was noted, patients were treated with Mannitol 5ml/kg/dose every 6 hours for 48 hours. Patients were followed up periodically and reviewed after one year of treatment as per hospital protocol. CT or MRI were performed after one year. Lesion regression in CT was defined as disappearance of the lesions or 50% or more reduction in number or size of lesions.

### Ethical committee approval

Prior to the study, the institutional Ethics Committee at Manipal Teaching hospital, Pokhara, Nepal, gave ethical approval. The Research was conducted in accordance with the latest version of the Declaration of Helsinki. Data obtained were anonymous to protect patient privacy and confidentiality.

### Statistical methods

Data analysis was performed using SPSS version 18. Continuous data was presented as mean and SD and categorical data expressed as frequency, percentage and 95% confidence interval. Trend of the data was explored using graphs and Poisson regression. NCC and total seizures were considered a dependent variable while time in years was taken as an independent variable.

## Results

### Distribution of NCC cases along time

**Fig 1:**
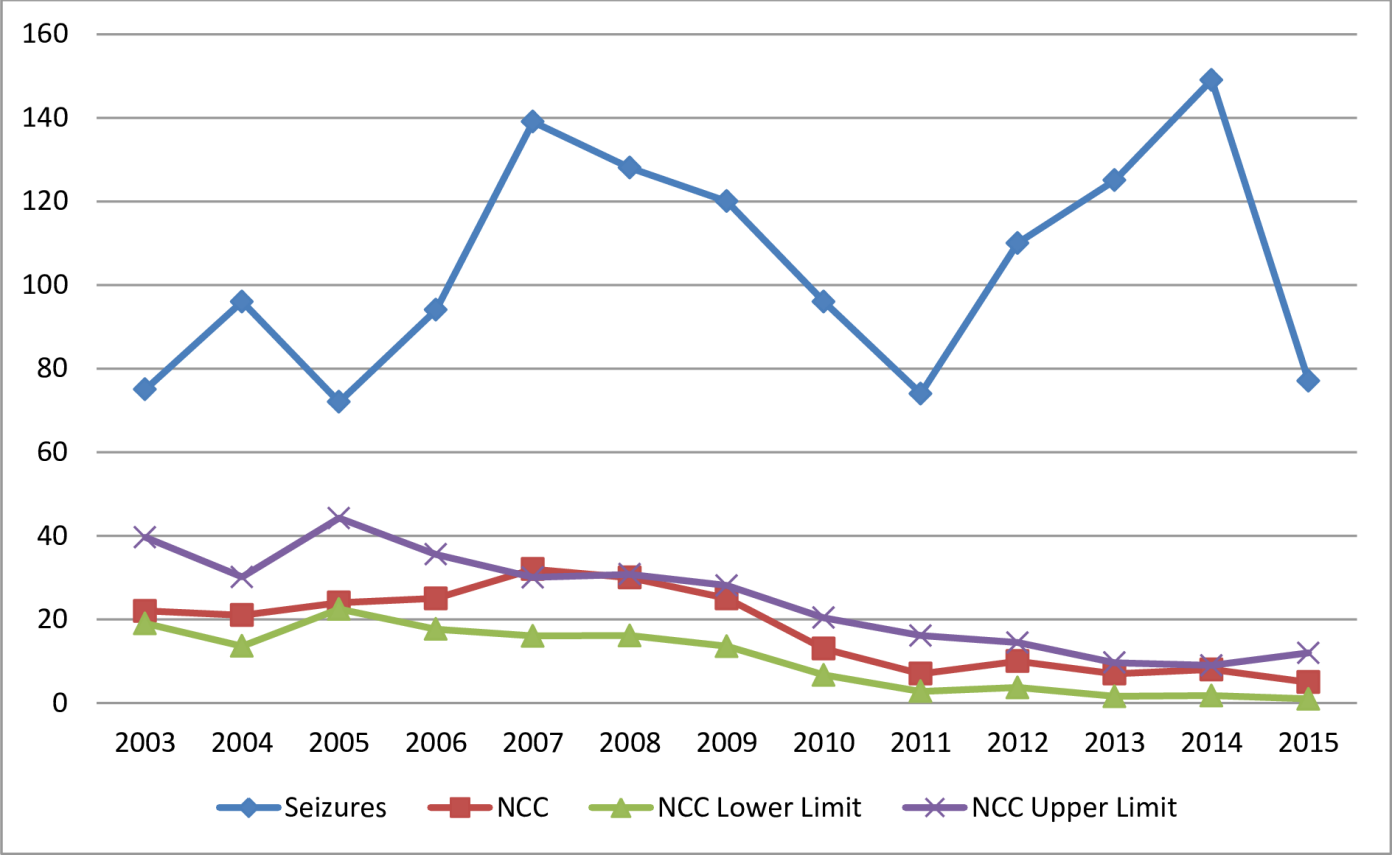
Distribution of NCC cases per year.

From January 1^st^ 2003 to December 31^st^ 2015, 1355 cases with seizure disorder were identified. Out of these, using clinical data and by CT and MRI, 229 cases were diagnosed as NCC (16.90%). The maximum number of NCC cases was detected in 2007 and 2008 (32 and 30 cases respectively). In 2005 out of 72 cases of seizure disorder, 24 were detected as NCC (Table 1). The incidence of NCC with seizures as presenting symptoms gradually decreased from 2009 to 2015 (20.83% to 5.37%). (Fig 1).

**Table 1:** Distribution of NCC cases per year.

Year	Seizures	NCC	Percentage	95% CI
2003	75	22	29.33	(19.03, 39.64)
2004	96	21	21.88	(13.61. 30.14)
2005	72	24	33.33	(22.44,44.22)
2006	94	25	26.60	(17.66,35.53)
2007	139	32	23.02	(16.02,30.02)
2008	128	30	23.44	(16.10,30.78)
2009	120	25	20.83	(13.57.28.10)
2010	96	13	13.54	(6.70,20.39)
2011	74	7	9.46	(2.79,16.13)
2012	110	10	9.09	(3.72,14.46)
2013	125	7	5.60	(1.57,9.63
2014	149	8	5.37	(1.75,8.99)
2015	77	5	6.49	(0.99,12.00)
	1355	229	16.90	(14.90,18.90)

Although the percent of cases slightly augmented in 2005, the general tendency between 2003 and 2015 shows a significant decrease in the number of cases and the percentage of epileptic patients with NCC, that was confirmed by Poisson regression analysis [B = -0.112, P = 0.0001] while there was minimal increase in the epileptic patients during the period [B = 0.020, P<0.007].

### Demographic, ethnic and district distribution

Mean age of incidence of NCC was 9.7± 3.44 years with median age of 10 years. Youngest age was 11 months and the oldest patient was 17 years. Maximum incidence of NCC presenting as seizure disorder was detected in the age group of 6–10 years (99/229, 43.23%) followed by 10–15 years (40.61%). Overall 192 cases were detected in age group of 6–15 years accounting for 83.84%. Male to female ratio is 6:4. According to ethnicity the maximum incidence was in Brahamins (30%) followed by Dalits (22%) and Chetris (20%); while according to district, the disease was more prevalent in Kaski (38%) followed by Syangja (36%) and Tanahun (10%); the remaining places had an incidence between 1–5%.

### Modes of presentation and sites of lesion

Out of 229 cases of NCC, 203 (88.65%) children presented with seizure as chief complaint; Simple partial seizures accounted for 140 cases (68.96%) followed by generalized seizures in 33 cases (16.20%) and complex partial seizures 30 (14.78%). A total of 38 cases of NCC (16.59%) presented as neuropsychiatry illness, 22 cases had raised intracranial pressure (ICP, 9.6%), 8 cases (3.49%) encephalitis and 3 ophthalmoplegia (1.31%). Fever was present in 8 cases (3.49%) (Table 2).The cases of raised ICP and encephalitis although not presented as seizure disorder, had seizures during management. Two cases of extra-parenchymal lesions also presented seizures.

**Table 2:** Clinical features of NCC.

Features	NCC	Percentage	95% CI
Seizures	203	88.65	(84.54,92.76)
1. Simple partial	140	68.97	(62.60,75.33)
2. Complex partial	30	14.78	(9.90,19.66)
3. Generalized	33	16.26	(11.18,21.33)
Features of raised ICP	22	9.61	(5.79,13.42)
Encephalitis	8	3.49	(1.12,5.97)
Prolonged headache with loss of consciousness	3	1.31	(0,2.78)
Ophthalmoplegia	3	1.31	(0,2.78)
Fever	8	3.49	(1.12,5.87)
Nuero Psychiatric Manifestations	38	16.59	(11.78,21.41)

**Fig 2:**
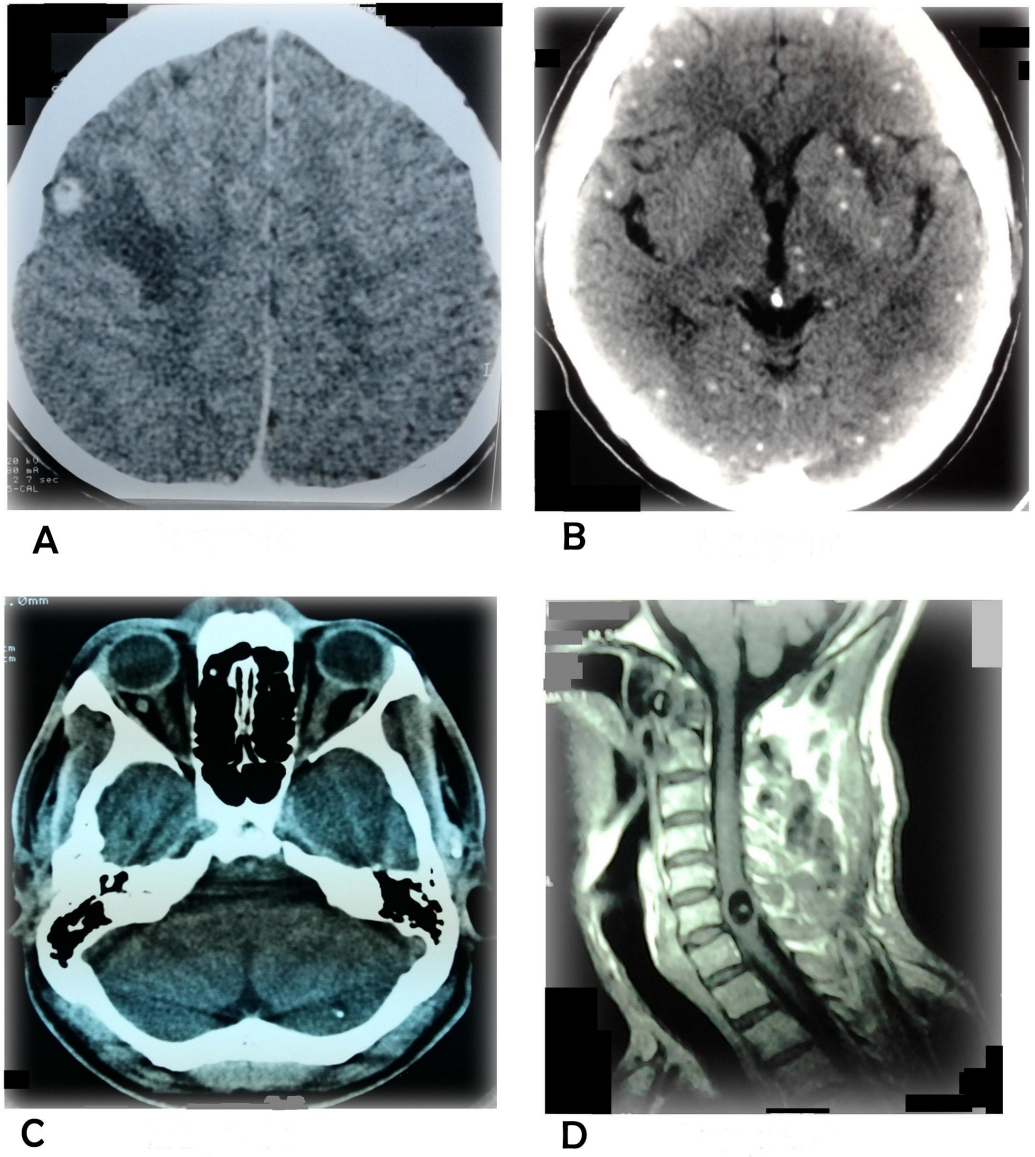
A-Single NCC with peri-lesional edema detected by CT in the right fronto-parietal area. B-Multiple NCC with starry sky pattern. C-Ocular NCC. D-Spinal NCC.

CT and MRI findings revealed a single NCC lesion in 179 cases (75.98%) and multiple NCC lesions in 65 cases (24.01%, Fig 2A and 2B), The site of involvement detected by CT (Table 3) varied and the lesions were located mainly in the parietal region (106 cases, 46.29%) followed by the frontal region (36, 15.72%), temporal (28, 12.27%) and occipital (24, 10.48%). Cerebellar lesions were found in 16 cases (7%), intraventricular in 14 (6.11%), 5 brain stem lesions (2.46%) and 2 cases each of subarachnoid and spinal lesions. Only 2 ocular lesions were seen. (Fig 2C and 2D) and 23 extraparenchymal lesions (10.04%).

**Table 3:** CT findings.

Site of Lesion	Number	Percentage	95% CI
Parietal	106	46.29	(39.83,52.75)
Frontal	36	15.72	(11.01,20.43)
Temporal	28	12.23	(7.98,16.47)
Occipital	24	10.48	(6.51,14.45)
Cerebellar	16	6.99	(3.69,10.29)
Intravetricular	14	6.11	(3.01,9.22)
Brain Stem	5	2.18	(0.29,4.08)
Subarachnoid	5	2.18	(0.29,4.08)
Ocular	2	0.87	(0,2.08)
Spinal	2	0.87	(0,2.08)

### Clinical outcome

Among 229 patients with NCC, 190 (82.97%) could be re-evaluated with a repeated CT at one year; the remaining 39 cases were lost for follow up. In the 190 cases reviewed, improvement was observed in 156 cases (82.10%) while in 2 cases (1.05%) lesions persisted and 32 cases (16.84%) had calcifications.

## Discussion

Cysticercosis is mainly divided into parenchymal and extra-parenchymal including cisternal, ventricular, ophthalmic or spinal lesions. The presentation is either as a single parenchymal cyst or multiple cysts. Seizures were mainly seen in parenchymatous location, although extra-parenchymatous lesions could also present seizures due to association with parenchymatous ones. [13]. Cysticercosis remains asymptomatic in the host for years as parasites evade host immunological responses [14].

Poisson regression analysis showed statistically significant year decline of NCC (p<0.05) from 2003 to 2015 in contrast with the marginal increase in the total number of children admitted with seizures during the study period. This indicated that although hospital admission due to NCC were reduced, total hospital admissions due to seizures or other etiologies like epilepsy, CNS infections, genetic conditions, metabolic derangements and structural malformations had increased. Out of 229 cases, 203 presented with sudden onset seizure (88.65%). The remaining 26 cases who presented with features of raised ICP, encephalitis, prolonged headache with loss of consciousness, had also manifested seizures during the course of management. In a series of 500 children from northern India, seizures were noted in 94.8% cases [14].

Partial seizures were common in children with NCC compared with generalized seizures in the current study. Earlier studies also described partial seizures in most of children with NCC [14–23] Features of raised ICP were seen in 22 cases (9.61%). Features of raised ICP was detected in 30% of patients by Singhi et al [14]. Clinical features suggestive of encephalitis were observed in 8 out of 229 cases (3.49%) in the present study. similar to incidence, ranging up to 4.8% was noted in earlier studies [21,22,23]; high morbidity and mortality can be associated [24]. NCC should be a major differential diagnosis in patient with seizure disorder [22]. Immunodiagnosis and histopathological studies of the lesions obtained by surgery, CT and MRI remains valuable diagnostic tools [23].

CT studies and, when necessary MRI, revealed diagnosis of NCC in 229 cases out of 1355 cases reviewed in the study. CT revealed single lesions in 174 cases (75.96%), 55 cases (24.01%) of multiple lesions and 90% of the cases revealed peri-lesional edema. Similar data were obtained by Shrestha et al, who described single lesions in 83.8% and peri-lesional edema in 89.7% of the cases [25]. Maximum number of NCC lesions were seen in the parietal region (46.29%) followed by the frontal region (15.72%), temporal (12.23%), occipital (10.48%) and lastly cerebellar region of the brain (6.99%); while Basu et al reported parietal (71%), frontal (49.2%), occipital (37.1%) and temporal (29.8%) as common sties in children [22]. Silva et al described isolated or associated calcifications in 95% of adult patients with NCC [26]. Extra-parenchymal lesions had manifested with the features of raised ICP, convulsions and altered sensorium. Other presentations were hydrocephalus, ophthalmoplegia and other focal deficiencies. Most patients with hydrocephalus had presented with features of encephalitis in our series.

Ethnical variations showed 30% of patients were Brahmins. Brahmins are the major ethnic group thus, demographic studies are necessary to identify other risk factors for NCC. A striking correlation between the use of cysticidal drugs and the rate of seizure control was found in adults with epilepsy due to NCC [17,27,28]. It was also found that focal neurological deficits improved after treatment with cysticidal drug. Among patients with complete disappearance of lesions and without repeated seizures, antiepileptic medication could be stopped at one year in 120 (63.1%). In 38 (20%) patients with delayed resolution of lesions, calcified lesions and recurrent seizures, antiepileptic medication was required for 2–3 years. The remaining 32 (16.8%) were still on antiepileptic medications beyond 3 years. Two children stopped attending school due to poor academic capacity. An improved outcome was reported in 80% cases in which treatment was given soon after recognition of a Single Small Enhancing Computer Tomographic Lesion [20].

NCC is a disease perpetuated by poor hygiene and inadequate sanitation; it is entirely preventable and potentially eradicable [29]. Risk factors for *T*. *solium* suggests that consumption of the raw meat, improper or absence of meat inspection and control, poor sanitation, and use of untreated human waste as fertilizer for crops could have played important roles in endemic areas [30]. An ideal intervention program to stop transmission should include the new proposal of "one health" that involves humans and pigs within the same environment. Studies from hyper-endemic areas showed that after a one-health intervention program (triple dose of 400mg Albendazole in the form of two mass drug administration) covering all village residents >6 years of age, the level of taeniasis decreased by 79.4%. After the first regime it remained steady with 5 month inter-treatment interval and decreased again by 100% after the second mass treatment regime [31]. A prospective interventional cohort controlled study using screening and treatment of taeniasis among households located within 100m of pigs heavily infested with cysticercus caused 41% reduction in incidence and four times reduction in prevalence of NCC in the intervention village as compared to a control village in northern Peru [32]. Preventive measures included avoidance of open defecation, improved food-handling practices such as thorough cleaning of raw vegetables prior to use, wearing gloves by food handlers and mass administration of anthelminthic drugs in endemic areas [33,34].

The present study covering the period of 2003 to 2015 revealed NCC as the main cause of seizure disorder and accounted for 16.9% of the patients presenting as simple, partial or generalized seizures; interestingly the rate of incidence of NCC as cause of seizures gradually declined from 20.83% in the year 2009 to 5.37% in 2014. In 2014 and 2015 only 13 cases of NCC were detected accounting for 5.65% of seizure cases. Improvement of health facilities like hand washing and construction of toilets in school, health education might have contributed in reduction in the incidence of NCC cases. The role of School Health Nutrition Program by the Child Health Division, under the Department of Health Services of the Ministry of Health and Population with biannual distribution of deworming tablets (Albendazole 400 mg) on NCC, needs further evaluation [35].

## Conclusions

Neurocysticercosis is one of the main causes of seizures in children of western Nepal accounting to 16.9% of the patients. In the present study children in the age group of 6–15 years (mostly school going children) accounted for 83.84% of 229 cases of NCC detected by seizure disorder, clinical and imaging techniques. Poisson regression analysis showed a statistically significant decline in the incidence of NCC cases from 2003 to 2015. This result is very interesting and is probably due to improvement of health facilities like hand washing and construction of toilets in schools and houses; Nonetheless implementation of the School Health Nutrition Program by the Child Health Division, under the Department of Health Services of Ministry of Health and Population was probably very important and should be further evaluated together with improved health education and sanitary infrastructure, which might have also contributed to the reduction in the incidence of NCC in children in Nepal.
